# 3′-[(1*H*-Indol-3-yl)carbon­yl]-1′-methyl-2-oxo-4′-(thio­phen-2-yl)­spiro­[indoline-3,2′-pyrrolidine]-3′-carbo­nitrile

**DOI:** 10.1107/S1600536813025890

**Published:** 2013-09-25

**Authors:** S. Antony Inglebert, Yuvaraj Arun, K. Sethusankar, Paramasivam T. Perumal

**Affiliations:** a Sri Ram Engineering College, Chennai 602 024, India; b Organic Chemistry Division, CSIR Central Leather Research Institute, Chennai 600 020, India; c Department of Physics, RKM Vivekananda College (Autonomous), Chennai 600 004, India

## Abstract

In the title compound, C_26_H_20_N_4_O_2_S, the central pyrrolidine ring adopts a twist conformation on the C—C bond involving the spiro C atom. Its mean plane makes dihedral angles of 78.83 (14), 65.91 (15) and 44.49 (18)° with the mean planes of the adjacent oxindole ring system, the indole system and the thio­phene ring, respectively. The indole and indoline units are essentially planar, with maximum deviations of 0.019 (3) and 0.090 (3) Å, respectively. In the oxindole fused-ring system, the pyrrole ring adopts an envelope conformation with the spiro C atom as the flap. In the crystal, pairs of N—H⋯O hydrogen bonds link the mol­ecules, forming inversion dimers with an *R* ^2^_2_(8) ring motif. The dimers are linked by further N—H⋯O hydrogen bonds, forming a two-dimensional network lying parallel to (100).

## Related literature

For background to indole derivatives and their biological activity, see: Srivastava *et al.* (2011 ▶). For puckering parameters, see: Cremer & Pople (1975 ▶). For bond-length data, see: Allen *et al.* (1987 ▶). For graph-set notation, see: Bernstein *et al.* (1995 ▶). For a related structure, see: Inglebert *et al.* (2013 ▶).
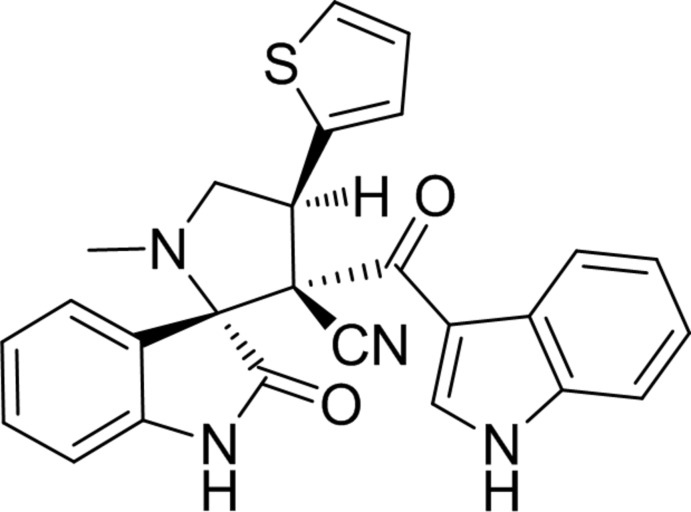


## Experimental

### 

#### Crystal data

C_26_H_20_N_4_O_2_S
*M* *_r_* = 452.52
Monoclinic, 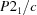

*a* = 11.6215 (6) Å
*b* = 17.1050 (11) Å
*c* = 12.3381 (8) Å
β = 114.431 (2)°
*V* = 2233.0 (2) Å^3^
*Z* = 4
Mo *K*α radiation
μ = 0.18 mm^−1^
*T* = 293 K
0.35 × 0.30 × 0.25 mm

#### Data collection

Bruker APEXII2 CCD difractometer diffractometer
Absorption correction: multi-scan (*SADABS*; Bruker, 2008 ▶) *T*_min_ = 0.941, *T*_max_ = 0.957
16319 measured reflections
5279 independent reflections
3636 reflections with *I* > 2σ(*I*)
*R*_int_ = 0.027

#### Refinement

*R*[*F* ^2^ > 2σ(*F* ^2^)] = 0.073
*wR*(*F* ^2^) = 0.252
*S* = 1.07
5279 reflections
306 parameters
5 restraints
H atoms treated by a mixture of independent and constrained refinement
Δρ_max_ = 1.15 e Å^−3^
Δρ_min_ = −0.98 e Å^−3^

### 

Data collection: *APEX2* (Bruker, 2008 ▶); cell refinement: *SAINT* (Bruker, 2008 ▶); data reduction: *SAINT*; program(s) used to solve structure: *SHELXS97* (Sheldrick, 2008 ▶); program(s) used to refine structure: *SHELXL97* (Sheldrick, 2008 ▶); molecular graphics: *Mercury* (Macrae *et al.*, 2008 ▶); software used to prepare material for publication: *SHELXL97* and *PLATON* (Spek, 2009 ▶).

## Supplementary Material

Crystal structure: contains datablock(s) global, I. DOI: 10.1107/S1600536813025890/su2642sup1.cif

Structure factors: contains datablock(s) I. DOI: 10.1107/S1600536813025890/su2642Isup2.hkl

Additional supplementary materials:  crystallographic information; 3D view; checkCIF report

## Figures and Tables

**Table 1:** Hydrogen-bond geometry (Å, °)

*D*—H⋯*A*	*D*—H	H⋯*A*	*D*⋯*A*	*D*—H⋯*A*
N3—H3*A*⋯O2^i^	0.84 (4)	2.12 (5)	2.828 (3)	142 (5)
N4—H4*A*⋯O1^ii^	0.85 (3)	1.99 (3)	2.829 (3)	177 (3)

Symmetry codes: (i) 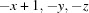; (ii) 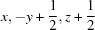.

**Fig. 1.**
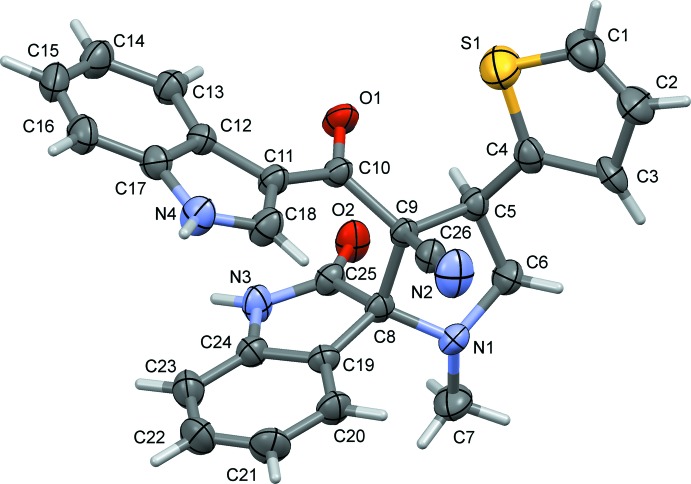
The molecular structure of the title molecule, with atom labelling. Displacement ellipsoids are drawn at the 30% probability level.

**Fig. 2.**
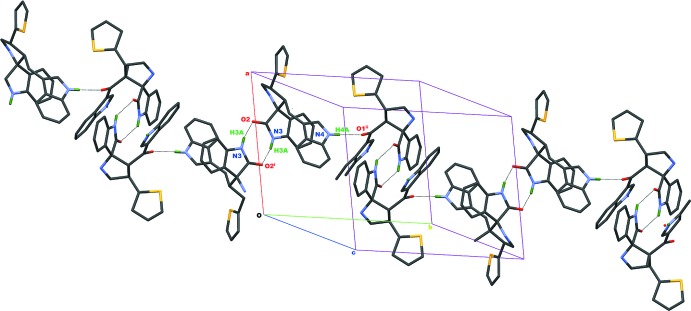
The partial view of thecrystal packing of the title compound along the c axis. Hydrogen bonds are shown as dashed lines (see Table 1 for details; H atoms not involved in hydrogen bonding have omitted for clarity).

## supplementary crystallographic information

### 1. Comment

The name indole is portmanteau of the words *indigo* and *oleum*, since indole was first isolated by treatment of the indigo dye with oleum. The indole compound is aromatic and solid at room temperature, and it has many applications in the fragrance and pharmaceutical industries. Several natural alkaloids having indole as their basic ring and have been found to be therapeutically active. The biological activities of indole derivatives include antimicrobial, antibiotic, analgesic, anticonvulsant, antimalarial, anticancer, antiulcer and antileishmanial (Srivastava *et al.*, 2011).

In the title compound, fig. 1, the central pyrrolidine ring adopts a *twisted* conformation on bond C8-C9 with puckering parameters (Cremer and Pople, 1975) of q(2) = 0.407 (3)Å and φ(2) = 133.5 (4)°. Atoms C8 and C9 deviate from the mean plane of the other atoms by 0.253 (3)Å and -0.232 (3)Å, respectively. The dihedral angle between the mean plane of the indole and the oxindole unit is 34.22 (11)°. The angles between the mean planes of the pyrrolidine ring and the oxindole and indole units are 78.83 (14)° and 65.91 (15)°, respectively.

The thiophene ring is perpendicular to the indole unit with a dihedral angle of 89.02 (15)°. Due to steric forces caused by the bulky substituents on the pyrrolidine moiety, the N—C and C—C bond lengths are slightly longer than the normal values. The sum of the angle around atom N1 (339.1 (7)°) is in accordance with sp^3^ hybridization. The indole and oxindole units are planar with maximum deviations of 0.019 (3)Å and -0.090 (3)Å for the atoms C11 and C8 from the mean planes. The thiophene ring is also essentially planar with a maximum deviation of -0.006 (4)Å for C2 atom.

The cyano bond distance C26≡N2 agrees well with the reported value of 1.138 (7) Å (Allen *et al.*, 1987). The small tilts between the planes of the five- and six-membered rings in the indole and oxindole units are 3.87 (16)° and 1.56 (18)°, respectively. Both the indole and oxindole moieties are individually quite planar and the dihedral angle between them is 34.22 (11)°. The title compound exhibits structural similarities with the previously reported related structure (Inglebert *et al.*, 2013).

Atom O1 deviates by 1.4756 (22) Å and 0.3082 (24) Å from the mean plane of the pyrrolidine ring and indole unit, respectively. Atom O2 attached to C25 is coplanar with the indoline ring system as indicated by the torsion angle O2–C25–N3–C24 = 175.5 (3)°.

In the crystal, molecules are linked by pairs of N—H···O hydrogen bonds, forming inversion dimers with an R^2^_2_(8) ring motifs (Bernstein *et al.*, 1995). The dimers are linked by N—H···O hydrogen bonds forming a two-dimensional network lying parallel to (100) [Table 1 and Fig. 2].

### 2. Experimental

A mixture of isatin (1 mmol), sarcosine (1.2 mmol) and 2-(1*H*-indole- 3-carbonyl)-3-(thiophen-2-yl)acrylonitrile (1 mmol) were refluxed in ethanol (30 ml). After completion of the reaction, as evidenced by TLC analysis, the reaction mixture was poured into ice-water. The resulting solid was filtered off and purified by column chromatography using ethyl acetate: petroleum ether (6:4) as an eluent to afford pure product. Slow evaporation of the solvents yielded block-like colourless crystals of the title compound.

### 3. Refinement

The H atoms were localized from the difference electron density maps. The N bound H atoms were freely refined. The C bound H atoms were treated as riding atoms: C-H = 0.93, 0.97 and 0.96 Å for CH, CH_2_ and CH_3_ H atoms, respectively, with U_iso_(H) = 1.5*U*_eq_(C-methyl) and = 1.2*U*_eq_(C) for other H atoms. The rotation angles for methyl groups were optimized by least squares.

### Crystal data

**Table:**

C_26_H_20_N_4_O_2_S	*F*(000) = 944
*M**_r_* = 452.52	*D*_x_ = 1.346 Mg m^−^^3^
Monoclinic, *P*2_1_/*c*	Mo *K*α radiation, λ = 0.71073 Å
Hall symbol: -P 2ybc	Cell parameters from 5279 reflections
*a* = 11.6215 (6) Å	θ = 2.2–27.9°
*b* = 17.1050 (11) Å	µ = 0.18 mm^−^^1^
*c* = 12.3381 (8) Å	*T* = 293 K
β = 114.431 (2)°	Block, colourless
*V* = 2233.0 (2) Å^3^	0.35 × 0.30 × 0.25 mm
*Z* = 4	

### Data collection

**Table:**

Bruker APEXII2 CCD difractometer diffractometer	5279 independent reflections
Radiation source: fine-focus sealed tube	3636 reflections with *I* > 2σ(*I*)
Graphite monochromator	*R*_int_ = 0.027
ω and φ scan	θ_max_ = 27.9°, θ_min_ = 2.2°
Absorption correction: multi-scan (*SADABS*; Bruker, 2008)	*h* = −15→8
*T*_min_ = 0.941, *T*_max_ = 0.957	*k* = −21→22
16319 measured reflections	*l* = −14→16

### Refinement

**Table:**

Refinement on *F*^2^	Primary atom site location: structure-invariant direct methods
Least-squares matrix: full	Secondary atom site location: difference Fourier map
*R*[*F*^2^ > 2σ(*F*^2^)] = 0.073	Hydrogen site location: inferred from neighbouring sites
*wR*(*F*^2^) = 0.252	H atoms treated by a mixture of independent and constrained refinement
*S* = 1.07	*w* = 1/[σ^2^(*F*_o_^2^) + (0.1409*P*)^2^ + 1.6573*P*] where *P* = (*F*_o_^2^ + 2*F*_c_^2^)/3
5279 reflections	(Δ/σ)_max_ = 0.003
306 parameters	Δρ_max_ = 1.15 e Å^−^^3^
5 restraints	Δρ_min_ = −0.98 e Å^−^^3^

### Special details

**Table:**

Geometry. All e.s.d.'s (except the e.s.d. in the dihedral angle between two l.s. planes) are estimated using the full covariance matrix. The cell e.s.d.'s are taken into account individually in the estimation of e.s.d.'s in distances, angles and torsion angles; correlations between e.s.d.'s in cell parameters are only used when they are defined by crystal symmetry. An approximate (isotropic) treatment of cell e.s.d.'s is used for estimating e.s.d.'s involving l.s. planes.
Refinement. Refinement of *F*^2^ against ALL reflections. The weighted *R*-factor *wR* and goodness of fit *S* are based on *F*^2^, conventional *R*-factors *R* are based on *F*, with *F* set to zero for negative *F*^2^. The threshold expression of *F*^2^ > σ(*F*^2^) is used only for calculating *R*-factors(gt) *etc*. and is not relevant to the choice of reflections for refinement. *R*-factors based on *F*^2^ are statistically about twice as large as those based on *F*, and *R*- factors based on ALL data will be even larger.

### Fractional atomic coordinates and isotropic or equivalent isotropic displacement parameters (Å^2^)

**Table:**

	*x*	*y*	*z*	*U*_iso_*/*U*_eq_	
C5	0.9175 (3)	0.04509 (16)	0.1198 (3)	0.0313 (6)	
H5	0.8526	0.0371	0.0390	0.038*	
C6	0.9536 (3)	−0.03507 (18)	0.1780 (3)	0.0403 (7)	
H6A	0.9204	−0.0760	0.1188	0.048*	
H6B	1.0448	−0.0403	0.2170	0.048*	
C7	0.8686 (3)	−0.12000 (19)	0.2877 (4)	0.0502 (8)	
H7A	0.9413	−0.1529	0.3064	0.075*	
H7B	0.7999	−0.1397	0.2180	0.075*	
H7C	0.8446	−0.1197	0.3534	0.075*	
C8	0.7970 (2)	0.01482 (16)	0.2360 (2)	0.0289 (6)	
C9	0.8532 (2)	0.08688 (16)	0.1927 (2)	0.0277 (6)	
C10	0.7470 (2)	0.14477 (16)	0.1152 (2)	0.0303 (6)	
C11	0.6903 (3)	0.19866 (17)	0.1672 (2)	0.0323 (6)	
C12	0.5730 (2)	0.24011 (16)	0.1035 (2)	0.0311 (6)	
C13	0.4806 (3)	0.24014 (19)	−0.0133 (3)	0.0394 (7)	
H13	0.4882	0.2079	−0.0706	0.047*	
C14	0.3773 (3)	0.2892 (2)	−0.0420 (3)	0.0474 (8)	
H14	0.3153	0.2895	−0.1196	0.057*	
C15	0.3641 (3)	0.3378 (2)	0.0420 (3)	0.0479 (8)	
H15	0.2946	0.3710	0.0192	0.058*	
C16	0.4511 (3)	0.33779 (19)	0.1576 (3)	0.0446 (8)	
H16	0.4413	0.3694	0.2145	0.054*	
C17	0.5550 (3)	0.28865 (18)	0.1871 (3)	0.0361 (6)	
C18	0.7336 (3)	0.22322 (19)	0.2844 (3)	0.0419 (7)	
H18	0.8071	0.2055	0.3463	0.050*	
C19	0.7534 (2)	0.03331 (17)	0.3323 (2)	0.0313 (6)	
C20	0.8205 (3)	0.0492 (2)	0.4520 (3)	0.0405 (7)	
H20	0.9083	0.0468	0.4870	0.049*	
C21	0.7536 (4)	0.0689 (2)	0.5186 (3)	0.0520 (9)	
H21	0.7974	0.0807	0.5989	0.062*	
C22	0.6227 (3)	0.0714 (2)	0.4675 (3)	0.0519 (9)	
H22	0.5799	0.0856	0.5136	0.062*	
C23	0.5554 (3)	0.0530 (2)	0.3496 (3)	0.0461 (8)	
H23	0.4676	0.0533	0.3156	0.055*	
C24	0.6223 (3)	0.03431 (18)	0.2832 (3)	0.0344 (6)	
C25	0.6696 (2)	−0.00651 (17)	0.1305 (2)	0.0320 (6)	
C26	0.9511 (3)	0.12412 (17)	0.2976 (3)	0.0338 (6)	
N1	0.8989 (2)	−0.04069 (14)	0.2652 (2)	0.0314 (5)	
N2	1.0305 (3)	0.1513 (2)	0.3775 (3)	0.0536 (8)	
N3	0.5757 (2)	0.01076 (17)	0.1639 (2)	0.0384 (6)	
N4	0.6549 (3)	0.27593 (17)	0.2955 (2)	0.0432 (6)	
O1	0.7082 (2)	0.13787 (13)	0.00656 (18)	0.0420 (5)	
O2	0.65774 (19)	−0.03273 (14)	0.03448 (19)	0.0431 (6)	
C3	1.1633 (3)	0.07184 (19)	0.1643 (3)	0.0387 (7)	
H3	1.2042	0.0288	0.2100	0.046*	
C1	1.1410 (4)	0.1943 (2)	0.0621 (4)	0.0541 (9)	
H1	1.1683	0.2385	0.0356	0.065*	
C2	1.2182 (3)	0.1393 (2)	0.1252 (3)	0.0540 (9)	
H2	1.3045	0.1423	0.1450	0.065*	
C4	1.0225 (3)	0.09175 (19)	0.1099 (3)	0.0369 (6)	
S1	0.98893 (10)	0.17802 (6)	0.03333 (12)	0.0667 (4)	
H3A	0.4965 (19)	0.008 (4)	0.130 (5)	0.110 (19)*	
H4A	0.669 (4)	0.303 (2)	0.357 (3)	0.066 (13)*	

### Atomic displacement parameters (Å^2^)

**Table:**

	*U*^11^	*U*^22^	*U*^33^	*U*^12^	*U*^13^	*U*^23^
C5	0.0313 (13)	0.0332 (14)	0.0297 (14)	0.0053 (11)	0.0129 (11)	−0.0025 (12)
C6	0.0417 (15)	0.0360 (16)	0.0468 (18)	0.0096 (12)	0.0220 (14)	0.0045 (14)
C7	0.0495 (18)	0.0358 (17)	0.066 (2)	0.0044 (14)	0.0248 (17)	0.0119 (16)
C8	0.0236 (11)	0.0356 (14)	0.0229 (12)	0.0021 (10)	0.0048 (10)	0.0001 (11)
C9	0.0257 (11)	0.0327 (13)	0.0229 (12)	0.0042 (10)	0.0084 (10)	0.0002 (11)
C10	0.0318 (12)	0.0323 (14)	0.0248 (13)	0.0061 (11)	0.0095 (11)	0.0021 (11)
C11	0.0337 (13)	0.0346 (14)	0.0265 (13)	0.0070 (11)	0.0103 (11)	0.0001 (11)
C12	0.0318 (13)	0.0314 (14)	0.0314 (14)	0.0035 (11)	0.0142 (11)	0.0020 (11)
C13	0.0350 (14)	0.0453 (17)	0.0357 (16)	0.0043 (12)	0.0123 (13)	0.0011 (14)
C14	0.0303 (14)	0.055 (2)	0.0478 (19)	0.0043 (13)	0.0066 (13)	0.0085 (16)
C15	0.0341 (15)	0.0502 (19)	0.062 (2)	0.0124 (13)	0.0223 (15)	0.0099 (17)
C16	0.0448 (16)	0.0409 (17)	0.056 (2)	0.0098 (14)	0.0284 (16)	0.0002 (15)
C17	0.0386 (14)	0.0352 (15)	0.0368 (16)	0.0033 (12)	0.0180 (13)	−0.0011 (13)
C18	0.0439 (16)	0.0426 (17)	0.0344 (16)	0.0111 (13)	0.0114 (13)	−0.0040 (13)
C19	0.0306 (13)	0.0366 (15)	0.0249 (13)	0.0020 (11)	0.0096 (11)	0.0030 (11)
C20	0.0363 (14)	0.0528 (19)	0.0269 (14)	0.0078 (13)	0.0076 (12)	0.0031 (13)
C21	0.058 (2)	0.070 (2)	0.0266 (15)	0.0113 (18)	0.0167 (15)	0.0025 (16)
C22	0.056 (2)	0.068 (2)	0.0418 (19)	0.0141 (17)	0.0301 (17)	0.0036 (17)
C23	0.0346 (15)	0.060 (2)	0.0468 (19)	0.0058 (14)	0.0197 (14)	0.0023 (16)
C24	0.0307 (13)	0.0400 (15)	0.0305 (14)	0.0023 (11)	0.0106 (11)	0.0029 (12)
C25	0.0271 (12)	0.0350 (14)	0.0280 (14)	0.0000 (11)	0.0056 (11)	−0.0010 (12)
C26	0.0300 (13)	0.0369 (15)	0.0329 (15)	0.0036 (11)	0.0114 (12)	−0.0023 (12)
N1	0.0288 (11)	0.0308 (12)	0.0310 (12)	0.0057 (9)	0.0088 (10)	0.0044 (10)
N2	0.0381 (14)	0.065 (2)	0.0456 (16)	−0.0050 (13)	0.0052 (13)	−0.0178 (15)
N3	0.0242 (11)	0.0548 (16)	0.0309 (13)	0.0000 (11)	0.0061 (10)	−0.0052 (12)
N4	0.0483 (15)	0.0466 (15)	0.0318 (14)	0.0116 (12)	0.0135 (12)	−0.0089 (12)
O1	0.0500 (12)	0.0498 (13)	0.0247 (10)	0.0188 (10)	0.0139 (9)	0.0043 (9)
O2	0.0329 (10)	0.0571 (14)	0.0322 (11)	0.0007 (9)	0.0064 (9)	−0.0134 (10)
C3	0.0327 (11)	0.0462 (16)	0.0462 (17)	−0.0091 (12)	0.0255 (12)	−0.0036 (14)
C1	0.057 (2)	0.0451 (19)	0.069 (2)	−0.0063 (16)	0.0351 (19)	−0.0027 (18)
C2	0.0441 (18)	0.057 (2)	0.066 (2)	−0.0071 (15)	0.0274 (17)	0.0100 (18)
C4	0.0348 (11)	0.0456 (14)	0.0338 (15)	0.0016 (12)	0.0177 (11)	−0.0060 (10)
S1	0.0605 (6)	0.0559 (6)	0.0906 (9)	0.0067 (4)	0.0380 (6)	0.0152 (5)

### Geometric parameters (Å, º)

**Table:**

C5—C4	1.505 (4)	C16—C17	1.390 (4)
C5—C6	1.524 (4)	C16—H16	0.9300
C5—C9	1.560 (4)	C17—N4	1.379 (4)
C5—H5	0.9800	C18—N4	1.331 (4)
C6—N1	1.462 (4)	C18—H18	0.9300
C6—H6A	0.9700	C19—C20	1.382 (4)
C6—H6B	0.9700	C19—C24	1.388 (4)
C7—N1	1.457 (4)	C20—C21	1.385 (5)
C7—H7A	0.9600	C20—H20	0.9300
C7—H7B	0.9600	C21—C22	1.386 (5)
C7—H7C	0.9600	C21—H21	0.9300
C8—N1	1.442 (3)	C22—C23	1.372 (5)
C8—C19	1.506 (4)	C22—H22	0.9300
C8—C25	1.557 (3)	C23—C24	1.379 (4)
C8—C9	1.587 (4)	C23—H23	0.9300
C9—C26	1.469 (4)	C24—N3	1.401 (4)
C9—C10	1.562 (4)	C25—O2	1.220 (3)
C10—O1	1.231 (3)	C25—N3	1.349 (4)
C10—C11	1.430 (4)	C26—N2	1.135 (4)
C11—C18	1.385 (4)	N3—H3A	0.84 (2)
C11—C12	1.446 (4)	N4—H4A	0.844 (19)
C12—C13	1.396 (4)	C3—C2	1.492 (4)
C12—C17	1.405 (4)	C3—C4	1.528 (4)
C13—C14	1.385 (4)	C3—H3	0.9300
C13—H13	0.9300	C1—C2	1.310 (5)
C14—C15	1.386 (5)	C1—S1	1.676 (4)
C14—H14	0.9300	C1—H1	0.9300
C15—C16	1.365 (5)	C2—H2	0.9300
C15—H15	0.9300	C4—S1	1.708 (3)
			
C4—C5—C6	116.2 (2)	N4—C17—C16	129.9 (3)
C4—C5—C9	113.7 (2)	N4—C17—C12	107.3 (2)
C6—C5—C9	104.2 (2)	C16—C17—C12	122.8 (3)
C4—C5—H5	107.5	N4—C18—C11	110.1 (3)
C6—C5—H5	107.5	N4—C18—H18	124.9
C9—C5—H5	107.5	C11—C18—H18	124.9
N1—C6—C5	106.7 (2)	C20—C19—C24	119.8 (3)
N1—C6—H6A	110.4	C20—C19—C8	131.2 (3)
C5—C6—H6A	110.4	C24—C19—C8	109.0 (2)
N1—C6—H6B	110.4	C19—C20—C21	118.3 (3)
C5—C6—H6B	110.4	C19—C20—H20	120.8
H6A—C6—H6B	108.6	C21—C20—H20	120.8
N1—C7—H7A	109.5	C20—C21—C22	121.2 (3)
N1—C7—H7B	109.5	C20—C21—H21	119.4
H7A—C7—H7B	109.5	C22—C21—H21	119.4
N1—C7—H7C	109.5	C23—C22—C21	120.8 (3)
H7A—C7—H7C	109.5	C23—C22—H22	119.6
H7B—C7—H7C	109.5	C21—C22—H22	119.6
N1—C8—C19	116.8 (2)	C22—C23—C24	118.0 (3)
N1—C8—C25	116.9 (2)	C22—C23—H23	121.0
C19—C8—C25	101.2 (2)	C24—C23—H23	121.0
N1—C8—C9	100.7 (2)	C23—C24—C19	122.0 (3)
C19—C8—C9	115.0 (2)	C23—C24—N3	128.6 (3)
C25—C8—C9	106.4 (2)	C19—C24—N3	109.4 (2)
C26—C9—C5	109.0 (2)	O2—C25—N3	126.6 (3)
C26—C9—C10	113.1 (2)	O2—C25—C8	125.8 (2)
C5—C9—C10	112.5 (2)	N3—C25—C8	107.6 (2)
C26—C9—C8	108.5 (2)	N2—C26—C9	177.1 (3)
C5—C9—C8	101.5 (2)	C8—N1—C7	115.0 (2)
C10—C9—C8	111.6 (2)	C8—N1—C6	109.6 (2)
O1—C10—C11	121.4 (2)	C7—N1—C6	114.5 (3)
O1—C10—C9	116.5 (2)	C25—N3—C24	112.0 (2)
C11—C10—C9	121.9 (2)	C25—N3—H3A	134 (4)
C18—C11—C10	128.8 (3)	C24—N3—H3A	114 (4)
C18—C11—C12	105.8 (2)	C18—N4—C17	110.3 (3)
C10—C11—C12	125.3 (2)	C18—N4—H4A	125 (3)
C13—C12—C17	118.2 (3)	C17—N4—H4A	124 (3)
C13—C12—C11	135.3 (3)	C2—C3—C4	102.1 (3)
C17—C12—C11	106.5 (2)	C2—C3—H3	129.0
C14—C13—C12	118.7 (3)	C4—C3—H3	129.0
C14—C13—H13	120.7	C2—C1—S1	114.2 (3)
C12—C13—H13	120.7	C2—C1—H1	122.9
C13—C14—C15	121.6 (3)	S1—C1—H1	122.9
C13—C14—H14	119.2	C1—C2—C3	117.9 (3)
C15—C14—H14	119.2	C1—C2—H2	121.1
C16—C15—C14	121.2 (3)	C3—C2—H2	121.1
C16—C15—H15	119.4	C5—C4—C3	127.1 (3)
C14—C15—H15	119.4	C5—C4—S1	119.7 (2)
C15—C16—C17	117.5 (3)	C3—C4—S1	113.2 (2)
C15—C16—H16	121.3	C1—S1—C4	92.66 (17)
C17—C16—H16	121.3		
			
C4—C5—C6—N1	132.7 (3)	N1—C8—C19—C24	−136.3 (3)
C9—C5—C6—N1	6.8 (3)	C25—C8—C19—C24	−8.2 (3)
C4—C5—C9—C26	−41.6 (3)	C9—C8—C19—C24	106.0 (3)
C6—C5—C9—C26	85.8 (3)	C24—C19—C20—C21	−2.6 (5)
C4—C5—C9—C10	84.7 (3)	C8—C19—C20—C21	176.3 (3)
C6—C5—C9—C10	−147.9 (2)	C19—C20—C21—C22	1.1 (6)
C4—C5—C9—C8	−155.9 (2)	C20—C21—C22—C23	1.1 (6)
C6—C5—C9—C8	−28.5 (3)	C21—C22—C23—C24	−1.8 (6)
N1—C8—C9—C26	−74.4 (3)	C22—C23—C24—C19	0.3 (5)
C19—C8—C9—C26	52.0 (3)	C22—C23—C24—N3	177.2 (3)
C25—C8—C9—C26	163.2 (2)	C20—C19—C24—C23	1.9 (5)
N1—C8—C9—C5	40.3 (2)	C8—C19—C24—C23	−177.1 (3)
C19—C8—C9—C5	166.8 (2)	C20—C19—C24—N3	−175.5 (3)
C25—C8—C9—C5	−82.1 (2)	C8—C19—C24—N3	5.4 (3)
N1—C8—C9—C10	160.4 (2)	N1—C8—C25—O2	−44.9 (4)
C19—C8—C9—C10	−73.2 (3)	C19—C8—C25—O2	−172.9 (3)
C25—C8—C9—C10	38.0 (3)	C9—C8—C25—O2	66.6 (4)
C26—C9—C10—O1	139.7 (3)	N1—C8—C25—N3	136.3 (3)
C5—C9—C10—O1	15.6 (4)	C19—C8—C25—N3	8.3 (3)
C8—C9—C10—O1	−97.7 (3)	C9—C8—C25—N3	−112.1 (3)
C26—C9—C10—C11	−45.4 (4)	C19—C8—N1—C7	65.8 (3)
C5—C9—C10—C11	−169.4 (3)	C25—C8—N1—C7	−54.3 (3)
C8—C9—C10—C11	77.2 (3)	C9—C8—N1—C7	−169.0 (2)
O1—C10—C11—C18	−168.2 (3)	C19—C8—N1—C6	−163.6 (2)
C9—C10—C11—C18	17.1 (5)	C25—C8—N1—C6	76.3 (3)
O1—C10—C11—C12	10.7 (5)	C9—C8—N1—C6	−38.4 (3)
C9—C10—C11—C12	−164.0 (3)	C5—C6—N1—C8	20.8 (3)
C18—C11—C12—C13	−176.8 (3)	C5—C6—N1—C7	151.7 (3)
C10—C11—C12—C13	4.1 (5)	O2—C25—N3—C24	175.5 (3)
C18—C11—C12—C17	1.3 (3)	C8—C25—N3—C24	−5.7 (3)
C10—C11—C12—C17	−177.8 (3)	C23—C24—N3—C25	−176.9 (3)
C17—C12—C13—C14	1.5 (4)	C19—C24—N3—C25	0.3 (4)
C11—C12—C13—C14	179.5 (3)	C11—C18—N4—C17	0.5 (4)
C12—C13—C14—C15	0.0 (5)	C16—C17—N4—C18	179.4 (3)
C13—C14—C15—C16	−1.7 (5)	C12—C17—N4—C18	0.3 (4)
C14—C15—C16—C17	1.6 (5)	S1—C1—C2—C3	−1.0 (5)
C15—C16—C17—N4	−178.9 (3)	C4—C3—C2—C1	1.1 (4)
C15—C16—C17—C12	0.1 (5)	C6—C5—C4—C3	−7.7 (4)
C13—C12—C17—N4	177.5 (3)	C9—C5—C4—C3	113.2 (3)
C11—C12—C17—N4	−1.0 (3)	C6—C5—C4—S1	173.7 (2)
C13—C12—C17—C16	−1.6 (5)	C9—C5—C4—S1	−65.4 (3)
C11—C12—C17—C16	179.8 (3)	C2—C3—C4—C5	−179.4 (3)
C10—C11—C18—N4	177.9 (3)	C2—C3—C4—S1	−0.7 (3)
C12—C11—C18—N4	−1.1 (4)	C2—C1—S1—C4	0.5 (3)
N1—C8—C19—C20	44.8 (4)	C5—C4—S1—C1	179.0 (3)
C25—C8—C19—C20	172.9 (3)	C3—C4—S1—C1	0.2 (3)
C9—C8—C19—C20	−72.9 (4)		

### Hydrogen-bond geometry (Å, º)

**Table:**

*D*—H···*A*	*D*—H	H···*A*	*D*···*A*	*D*—H···*A*
N3—H3*A*···O2^i^	0.84 (4)	2.12 (5)	2.828 (3)	142 (5)
N4—H4*A*···O1^ii^	0.85 (3)	1.99 (3)	2.829 (3)	177 (3)

Symmetry codes: (i) −*x*+1, −*y*, −*z*; (ii) *x*, −*y*+1/2, *z*+1/2.
